# Letter to the editor concerning “The status of nuclear medicine in China: the first official national survey”

**DOI:** 10.1007/s00259-024-06829-0

**Published:** 2024-07-05

**Authors:** Yifan Dong, Shuo Zhang

**Affiliations:** Department of Nuclear Medicine, the First Affiliated Hospital of Dalian Medical University, No.222, Zhongshan Road, Xigang District, Dalian, 116011 Liaoning China

**Keywords:** Nuclear Medicine, China, Quality control

Dear Editor,

We greatly appreciated the article from the Li Huo team entitled “The status of nuclear medicine in China: the first official national survey” by Haiqiong Zhang et al. [1]. The survey presented detailed findings from the first official national assessment of nuclear medicine in China. The comprehensive data provided offers an invaluable snapshot of the current landscape, establishing a benchmark for future enhancements in this vital field.

The authors have meticulously outlined the distribution and capabilities of nuclear medicine resources across China, highlighting significant achievements and identifying areas needing enhancement, especially in terms of PET equipment and radiopharmaceutical quality control.

The data presented in this article, current as of 2020, highlighted variations in the frequency of PET equipment testing across medical institutions, with some institutions yet to implement quality control of radiopharmaceuticals for PET. Recent years have seen advancements in the quality control of PET equipment. The National Health Commission of the People’s Republic of China issued quality control standards for PET equipment on March 7, 2023, which were implemented on March 1, 2024 [2]. This standard meticulously delineates the requirements for PET quality control testing, specifying both the items to be tested and the intervals for these tests. For the stability test of PET equipment quality control, the two test items of spatial resolution and sensitivity must be tested every six months. Noise equivalent count rate, scatter fraction, accuracy (count loss and random coincidence correction), and time-of-flight resolution are recommended to be tested every six months if conditions permit. In addition, medical institutions should conduct detector operational status test at least weekly and the calibration factor test every six months according to the requirements of the equipment manufacturer, with clinical diagnostics only continuing after these tests are passed. The standards specify that quality control tests must be recorded. The implementation of the new standard will effectively improve the current situation of large differences in PET equipment quality control test rates in different hospitals, and avoid excessive and overly conservative quality control test for PET. With the establishment and enforcement of these standards, the quality control of PET equipment in China is expected to become more standardized, thereby enhancing the overall quality of PET diagnostics and treatment [3].

Additionally, the data presented in this article indicated that about one-third of the hospitals had not conducted quality control on radiopharmaceuticals, potentially compromising the safety and effectiveness of treatments. This issue may be linked to a shortage of specialized personnel and inadequacies in the relevant systems. The Nuclear Medicine Residency Training Program in China has been a cornerstone in the development of nuclear medicine, having trained approximately 2000 physicians since 2014 [4]. It is imperative to further strengthen the management of radiopharmaceutical quality control through the development or revision of pertinent policies and regulations.

This survey is a commendable first step towards a systematic enhancement of nuclear medicine services in China. It provides a foundation for policymakers and healthcare providers to strategize improvements and ensure equitable access to nuclear medicine across the country.

Sincerely,

Yifan Dong and Shuo Zhang.

## Data Availability

Not applicable.
